# Association Between the Proportion of Women on a Conference Planning Committee and the Proportion of Women Speakers at Medical Conferences

**DOI:** 10.1001/jamanetworkopen.2020.0677

**Published:** 2020-03-12

**Authors:** Kirstie C. Lithgow, Madalene Earp, Aleem Bharwani, Sarah Fletcher, Shannon M. Ruzycki

**Affiliations:** 1 Division of Endocrinology, Department of Medicine, Cumming School of Medicine, University of Calgary, Calgary, Alberta, Canada; 2 Department of Oncology, University of Calgary, Calgary, Alberta, Canada; 3 Division of General Internal Medicine, Department of Medicine, Cumming School of Medicine, University of Calgary, Calgary, Alberta, Canada; 4 Faculty of Medicine, University of British Columbia, Vancouver, British Columbia, Canada; 5 Department of Community Health Sciences, Cumming School of Medicine, University of Calgary, Calgary, Alberta, Canada

## Abstract

This cross-sectional study examines the association of the proportion of women on a medical conference planning committee with the proportion of women speakers at the conference.

## Introduction

Women physician representation at academic meetings is an important facet of gender equity^1^; however, women continue to be underrepresented.^2^ The inclusion of women on conference planning committees is proposed to increase the number of women speakers.^1,3^ We sought to assess the association of women’s representation on conference planning committees with the proportion of women speakers at medical conferences in North America.

## Methods

We performed a cross-sectional analysis examining the association of the proportion of women on a conference planning committee (ie, committee composition) with the proportion of women speakers at the conference (ie, speaker composition) for medical conferences held in 2017. Details of our search strategy and selection criteria have been previously reported.^2^

The data for this study were publicly available on conference websites; therefore, the institutional ethics review board of the University of Calgary waived the need for review and for obtaining participant informed consent. This report follows the Strengthening the Reporting of Observational Studies in Epidemiology (STROBE) reporting guidelines for cross-sectional studies.

We obtained names of the planning committee members from the conference program or website or by contacting the society’s administrative team. Gender was determined using the Gender Balance Assessment Tool.^4^ For meetings where this information was not publicly available, committee names or committee composition were requested from the conference administration.

Linear regression was used to examine the association between committee composition and speaker composition. Two-sided *P* values were generated using Satterthwaite approximations for degrees of freedom in lmerTest statistical software version 3.1 (R Project for Statistical Computing). *P* < .05 was considered statistically significant. We adjusted for the proportion of women in each specialty (specialty composition) using current population-based data^5,6^ in a linear regression and linear mixed model. In the linear mixed model, medical specialty was included as a grouping factor (random effect and random intercepts with fixed mean), reflecting the fact that some specialties have multiple conferences. Committee and specialty composition were modeled as fixed effects. Linear mixed models were fit using the function lmer from the lme4 package version 1.1-21 for R statistical software version 3.6.1 (R Project for Statistical Computing). Final data analyses were performed on August 18, 2019.

## Results

Speaker composition was known for 181 meetings held in 2017.^2^ Committee composition was determined for 106 meetings (48 did not respond, 7 declined, and 20 had no information available).

The mean (SD) proportion of women on the planning committee was 37.3% (19.4%) (range, 0%-93.4%) (Figure), slightly higher than the mean proportion of women speakers previously reported as 34.9%.^2^ Linear regression indicated that committee composition explains 36.0% of the variance in speaker composition (*R*^2^ = 0.36; *F*_1,104_ = 58.6; *P* < .001). Committee composition was statistically significantly associated with speaker composition (β [SE], 0.46 [0.06]; *P* < .001) such that for every 10% increase in proportion of women on a planning committee there was a mean (SE) 4.6% (0.6%) increase in proportion of women speakers.

**Figure.**
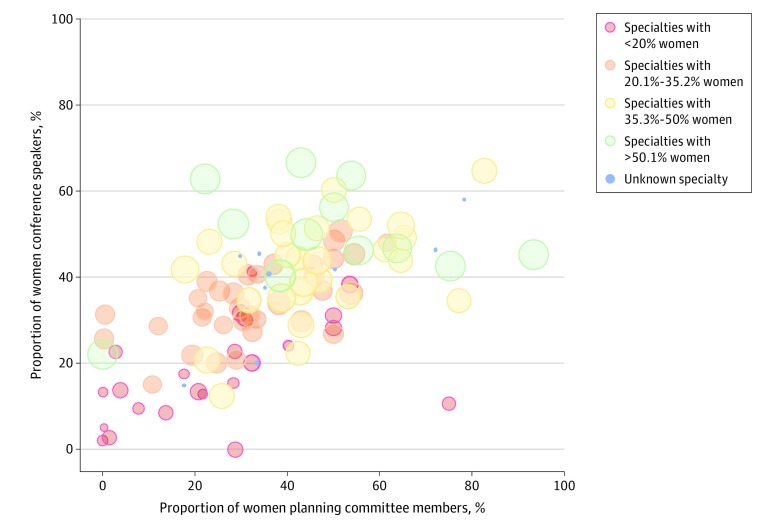
Proportion of Women Conference Speakers vs Proportion of Women Planning Committee Members According to Percentage of Women Members per Specialty

Adding specialty composition to the linear regression explained 56.8% of the variance in speaker composition (*R*^2^ = 0.57; *F*_2,94_ = 61.9; *P* < .001). Both committee composition (β [SE], 0.29 [0.06]; *P* < .001) and specialty composition (β [SE], 0.50 [0.07]; *P* < .001) independently were associated with speaker composition. Eleven conferences were omitted from the model because specialty composition could not be determined. After adjusting for gender composition of medical specialties, there was a mean (SE) 2.9% (0.6%) increase in the proportion of women speakers for every 10% increase in the proportion of women on the planning committee (Figure).

A linear mixed model was run to specify medical specialty as a grouping factor. This yielded similar estimates for the fixed effects coefficients. Committee composition was statistically significantly associated with speaker composition (β [SE], 0.28 [0.05]; *P* < .001), as was specialty composition (β [SE], 0.54 [0.08]; *P* < .001).

## Discussion

These findings suggest that a higher proportion of women on a planning committee is associated with greater proportions of women speakers, even after adjustment for specialty. Limitations of our study include its cross-sectional design (limiting assessment for confounders) and missing committee data from 75 meetings, which is a potential source of bias. Our findings are further supported by literature in nonmedical fields,^1,3^ which has demonstrated that involvement of women on planning committees is associated with greater representation of women speakers. Professional societies should commit to establishing gender balance within conference planning committees, because this may represent an opportunity to promote gender equity among program speakers.
